# Kidney Disease in HIV Infected Children on HAART in Nigeria

**DOI:** 10.4314/ahs.v25i1.11

**Published:** 2025-03

**Authors:** Marcia M Ihekaike, Maryam Shehu, Ikechukwu O Mbah, Patience U Kanhu, Hassan Shehu, Christian O Isichei

**Affiliations:** 1 Department of Paediatrics, Bingham University Teaching Hospital, Jos, Nigeria; 2 Department of Medicine, Nephrology division, Bingham University Teaching Hospital, Jos, Nigeria; 3 Department of Paediatrics, Jos University Teaching Hospital, Jos, Nigeria; 4 Department of Surgery, Paediatric Surgery Unit, Bingham University Teaching Hospital, Jos, Nigeria; 5 Department of Chemical Pathology, Jos University Teaching Hospital, Jos, Nigeria/Faith Alive Foundation, Jos

**Keywords:** HIV, kidney disease, HAART, Nigeria

## Abstract

**Background:**

In Nigeria, HIV/AIDS contributes to leading causes of morbidity and mortality and children living with HIV on HAART tend to live longer therefore, they are more likely to develop kidney diseases.

**Objective:**

This study aimed to evaluate the prevalence of kidney diseases in HIV-infected children and identify potential risk factors with a specific focus on antiretroviral regimens.

**Methods:**

A cross-sectional study conducted in a cohort of 121 consecutive children with HIV/AIDS on HAART. Serum creatinine level was determined and the estimated GFR was calculated using modified Schwartz formula. Kidney disease was defined as eGFR <90 mL/min/1.73 m2 and or dipstick proteinuria ≥1+.

**Results:**

This study included 65 (53.7%) females and 56 (46.3%) males with mean age of 10.8 ± 4.3 years. The children were all on antiretroviral medication. Proteinuria was observed in 7 (5.8%) of the participants, 6 (5.0%) had mild reduction of eGFR, and 1 (0.8%) each had moderately and severely reduced eGFR. A statistically significant association was observed between renal disease and high viral load, as well as the use of an abacavir or tenofovir-based regimen.

**Conclusion:**

These findings emphasize the importance of regular kidney function monitoring and tailored antiretroviral therapy selection in the management of HIV-infected children.

## Introduction

In 2021, of the predicted 38.4 million human immunodeficiency virus (HIV) positive individuals globally, 2.73 million were children under the age of 19.1. There was a predicted 850 paediatric HIV infections per day in 2021, as well as the approximately 301 paediatric acquired immune deficiency syndrome (AIDS) -related fatalities per day.^1^

Due to the destruction and impairment of immune cell functions caused by the human immunodeficiency virus, immune-deficient children are more susceptible to infections, chronic diseases, malignancies, and end-organ damage.^2^,^3^ A serious consequence of advanced HIV disease is renal impairment^6^ and children who have contracted HIV vertically are at a high risk of developing kidney disease.^7^

HIV-associated nephropathy (HIVAN) was the leading cause of chronic kidney disease (CKD) in children infected with HIV prior to widespread availability of antiretroviral therapy (ART).^4^,^8^ Being that antiretroviral (ARV) medications are now widely available, children living with HIV who are on Highly Active Anti-Retroviral Therapy (HAART) are now surviving for a comparatively longer period of time and as a result, likely to develop kidney diseases more frequently.^4^,^5^ This may be due to several reasons including use of HAART. In HIV-infected children and adolescents, ART, particularly tenofovir disoproxil fumarate (TDF), raises the incidence of proteinuria.^9^,^10^ Abacavir has been reported to cause impairment of kidney function as part of the abacavir hypersensitivity systemic clinical syndrome.^11^ Before any significant decline in glomerular filtration rate (GFR) was identified as a risk factor for the start and progression of kidney disease, proteinuria was described as the first sign of kidney disease in HIV infected patients.^12^-^14^

In Nigeria HIV/AIDS contributes to leading causes of morbidity and mortality in children. Kidney diseases among HIV-infected individuals have become increasingly recognized as a significant co-morbidity. Knowing the prevalence and predictors of kidney diseases in HIV-infected children allows for anticipation and screening of these individuals, which can lead to early detection and rapid initiation of therapy, thereby, lowering related morbidity and mortality. Therefore, this study aimed to investigate the prevalence of kidney diseases in HIV-infected children and explore potential associations with viral load and specific antiretroviral regimens.

## Methods

The study was carried out at the paediatric HIV clinic at Faith Alive Foundation Hospital and PMTCT Centre in Jos, Plateau State, North central, Nigeria between August and November, 2022. This was a cross sectional study that recruited one hundred and twenty-one consecutive children aged 17 years and below who were HIV positive and on HAART from the paediatric ART clinic of the Faith Alive Foundation Hospital and PMTCT Centre. This a secondary health care facility that caters for the needs of HIV infected individuals.

Consecutive sampling of all HIV infected children and adolescents who were on HAART and consented to the study was done. Excluded from the study were HIV infected children who were not on HAART or who did not consent to the study.

Ethical clearance was obtained from the hospital's Health Research Ethics Committee before the commencement of the study. Informed consent and assent (for children who have reached the age of assent which is 7 years) were obtained from each client and their care-givers before being recruited into the study.

Socio-demographic characteristics of participants including age and gender were obtained via a structured questionnaire. Parents/guardian level of education and occupation were obtained and used to classify participants into the socio-economic classes using the Olusanya et al method of classification. 15 A brief physical examination was performed and weight, height/length and blood pressure were recorded for each participant. The body mass index (BMI) was calculated using the participants weight and height. The children were classified according to the World Health Organisation (WHO) clinical staging of HIV into stages I to IV.^16^

The diagnosis of HIV infection was made using the Alere™ Determine HIV 1/2 and Trinity Biotech Uni-Gold™ HIV test kits. For children less than 2 years of age, the confirmation of HIV infection was made using the dried blood spot for DNA PCR test. The AMPLICOR® HIV-1 MONITOR test v1.5 (Roche Molecular Systems, Inc., Branchburg, NJ 08876, USA) was used to quantify HIV viral load (VL). The participants were categorized based on their viral load results into undetectable when VL ≤ 10 copies/ml, virally suppressed when VL = 11- 1000 copies/ml and unsuppressed when VL > 1000 copies/ml. 17

Serum creatinine was measured using the COBAS C 111 chemistry auto-analyzer. The serum creatinine was used to estimate the glomerular filtration rate (GFR) using the Schwartz formula.^18^

Urine analysis was done using the Medi-Test™ COMBI 10 dipstick test strips on freshly voided spot urine samples.

Kidney disease was defined as presence of dipstick proteinuria ≥1+, or reduced estimated GFR using serum creatinine (eGFRcr) of < 90ml/min/1.73m^2^

The data (age, gender, socio-economic class, status of the parents, weight, height, blood pressure, mode of HIV transmission, WHO clinical staging, viral load, serum creatinine, GFR) collected were entered manually into the computer and analysed using the Statistical Package for the Social Sciences (SPSS) version 27.0. Quantitative variables were expressed using means, median, and standard deviation as appropriate while qualitative variables were summarised using frequencies and percentages. Chi square test was used to test for relationships and a P-value of less than 0.05 was considered statistically significant in comparative analysis.

## Results

This study included 121 children with a male to female ratio of 1:1.16 with mean age of 10.8 ± 4.3 years with an age range of 1-17 years. 96.7% had vertical transmission of HIV infection, 85 (70.2%) had WHO stage 1 illness, 11 (10.3%) had unsuppressed VL, 59 (48.8%) were from low socioeconomic backgrounds (Table 2). All the patients had normal blood pressure. The mean systolic blood pressure (SBP) was 98.72 ± 9.735 and mean diastolic blood pressure (DBP) was 67.00 ± 9.571.

**Table 2:** Characteristics of study participants by degree of kidney disease based on eGFR_cr_

Variable	Moderate to Severe reduction in eGFR_cr_	Mild reduction in eGFR_cr_	Total No. with renal dysfunction	No renal disease	Total (%)	P value
**No. of participants**	2 (1.7)	6 (5.0)	8	113 (93.3)	121	
**Gender**						0.606
**Male**	0	3	3	53	56 (46.3)	
**Female**	2	3	5	60	65 (53.7)	
						0.007
**Age group (years)**			1	16	17 (14.0)	
**<** **6**	1	2	0	52	52 (43.0)	
**6 – 12** **>12**	0 1	0 6	7	45	52 (43.0)	
**sec**						0.120
**Upper**	0	3	3	17	20 (16.5)	
**Middle**	0	2	2	40	42 (34.7)	
**Lower**	2	1	2	56	59 (48.8)	
**Mode of HIV transmission**						0.864
**Vertical**	2	6	3	109	117 (96.7)	
**Horizontal**	0	0	0	4	4 (3.3)	
**Parents Alive**						0.443
**Yes**	1	5	6	65	71 (58.7)	
**No**	1	1	2	48	50 (41.3)	
**Duration of HIV infection (years)**						
<5	2	0	2	45	47 (38.8)	
≥5	0	6	8	68	74 (61.2)	
**WHO clinical staging**						0.573
**1**	1	5	6	79	85 (70.2)	
**2**	1	1	2	33	35 (29.0)	
**3**	0	0	0	1	1 (0.8)	
**Viral Load Category**						0.030
**Undetectable**	0	0	0	42	42 (39.2)	
**Virally suppressed**	1	6	7	47	54 (50.5)	
**Unsuppressed**	1	a	1	10	11 (10.3)	
**Tenofovir use On tenofovir**	1	6	7	43	50 (41.3)	0.006
**Not on tenofovir**	^1^	0	^1^	70	71 (58.7)	
**Abacavir use On abacavir**	1	0	1	67	68 (56.2)	0.004
**Not on abacavir**	1	6	7	46	53 (43.8)	

SEC = Socioeconomic Class, WHO = World Health Organisation

Proteinuria was observed in 7 (5.8%) of the participants all of whom had +1 protein. We found 6 (5.0%) who had mild reduction of eGFR, and 1 (0.8%) each who had moderately and severely reduced eGFR. Based on eGFR alone, age, duration of HIV infection, viral load, and use of tenofovir and abacavir were significantly associated with renal dysfunction (Table 2).

Only one participant had both proteinuria and some reduction in eGFR. When we combined participants that had both proteinuria and reduced eGFR, we found that a total of 14 (11.6%) of the children had some form of kidney dysfunction. Clinically, none of the children with renal dysfunction had symptoms suggestive of renal disease.

There was no significant relationship between the mean BMI of participants with reduction in eGFR (P = 0.17) (Table 3). The children were all on antiretroviral medication, 50 (41.3%) were on tenofovir-based regimen, and 68 (56.2)% were on abacavir.

**Table 3:** Mean and Standard Deviation of some Parameters of Participants by degree of kidney complications based on eGFR_cr_

Parameter	Moderate to Severe reduction in eGFR_cr_	Mild reduction in eGFR_cr_	No renal disease	P value
**No. of participants Mean BMI (kg/m^2^)**			113	
	20.2 ± 1.99	21.4 ± 2.18	17.4 ± 4.3	0.17
**Mean Systolic BP (mmHg)**	85.0 ± 21.21	98.33 ± 4.08	98.99 ± 9.66	0.87
**Mean Diastolic BP (mmHg)**	60.0 ± 14.14	63.33 ± 8.165	67.33 ± 9.58	0.32
**Mean Serum Cr (mg/dl)**	1.75 ± 0.35	0.80 ± 0.11	0.39 ± 0.13	<0.001
Mean GFR (mL/min/1.73m^2^*)	27.5 ± 17.68	78.17 ± 9.37	147.31 ± 50.45	<0.001

BMI = Body Mass Index, BP = Blood Pressure, Cr = Creatinine, GFR = Glomerular Filtration Rate

When proteinuria was combined with reduced eGFR, only viral load and abacavir based regimen were significantly associated with kidney disease (Table 4).

**Table 4:** Relationship between kidney disease (proteinuria and low eGFR) and some risk factors

Variable	df	OR (95% CI)	P- value
**WHO clinical staging**	2		0.138
**Viral load category**	2		0.019
**Duration of HIV infection**	1	2.56 (0.68 – 9.72)	0.155
**Mode of HIV transmission**	1	0.88 (0.82 – 0.94)	0.607
**Tenofovir based regimen**	1	0.35 (0.11 – 1.10)	0.064
**Abacavir based regimen**	1	3.72 (1.10 – 12.6)	0.027
**Protease inhibitor regimen**	1	1.05 (0.12 – 9.09)	0.721
**Dolutegravir regimen**	1	1.21 (0.24 – 6.00)	0.820

df = degree of freedom, WHO = World Health Organisation

## Discussion

The study's findings reveal that 11.6% of the examined children displayed renal dysfunction. Notably, factors such as age, viral load, duration of HIV infection and utilization of tenofovir and abacavir-based regimens demonstrated significant associations with this dysfunction.

Regarding specific renal markers, proteinuria was evident in 5.8% of the participants. Comparisons with rates documented elsewhere show both consistencies and disparities. Notably, the observed 5.8% proteinuria rate aligns with reported rates in Calabar (3.4%)^20^ and Tanzania (7.1%).^21^ However, our rate was notably lower than rates observed in Zimbabwe, Congo, and the United States of America.^5^,^22^-^23^ These divergent findings underscore the variability across populations and methodologies.

An analysis of glomerular filtration rate (GFR) revealed a reduction in 6.6% of participants, whereas a more concerning eGFRcr value below 60 ml/min/1.73m^2^ was found in only 1.7%. Interestingly, parallels can be drawn to similar studies conducted in sub-Saharan Africa (SSA)^24^ and the Caribbean^25^, which reported similarly low prevalence rates of 1.6% and 1.7%, respectively.

The overall prevalence of kidney disease among HIV-infected children, quantified at 11.6%, draws comparisons with rates documented elsewhere, including the 16.2% in Benin City, Nigeria.^26^ Notably, it also diverges from the higher 36.3% prevalence in Calabar^20^ and a staggering 48% prevalence in an Indian^27^ study. These divergent findings underline the pivotal role of standardized definitions of kidney dysfunction and the methodologies^29^ used for assessment across different research efforts.

The prevalence of kidney diseases in HIV-infected children observed in this study aligns with the existing understanding of the increased risk of renal complications in this population. Our investigation revealed that children who had been living with HIV for over 5 years exhibited a 2.56-fold increase in the likelihood of developing kidney diseases in comparison to those infected for a shorter duration. This observation raises the possibility of a progression towards kidney disease with extended HIV infection, corroborating earlier research findings.^6^,^30^ These outcomes underscore the importance of proactive and regular screening for kidney diseases in children living with HIV.

The association between renal dysfunction and high viral load is consistent with previous research, suggesting a possible link between uncontrolled viral replication and renal damage.^24^,^31^-^32^

Children who were prescribed an abacavir-based regimen had a 3.72-fold higher likelihood of developing kidney disease compared to their peers who were not using abacavir. The significant association between renal dysfunction and the use of an abacavir-based antiretroviral regimen raises important clinical implications. It is worthy of note however, that only 1 (1.5%) of the children on abacavir developed renal dysfunction, thus there may be other cofounders like the duration of HIV infection or viral load contributing to the occurrence of renal dysfunction in the child on abacavir. Abacavir is a commonly used nucleoside reverse transcriptase inhibitor (NRTI) in the management of HIV infection. While its efficacy in suppressing viral replication has been well-documented, emerging evidence has pointed to its potential nephrotoxic effects. The mechanism underlying this association requires further investigation but might involve direct renal cellular toxicity or immune-mediated responses.^33^

Though children who were prescribed tenofovir-based regimens had a mere 0.35-fold higher risk of developing renal issues than their counterparts who did not take tenofovir, we discovered a significant correlation between its use and the emergence of kidney disease, consistent with previous research findings.^8^,^34^-^36^ It's worth mentioning that both tenofovir and indinavir have been linked to the highest risk of kidney impairment in HIV-infected children.^8^

Children with HIV infection who are on ART, are likely to continue receiving treatment for the rest of their lives. These findings underscore the necessity for cautious consideration of antiretroviral choices in HIV-infected children. Clinicians should weigh the benefits of viral suppression against the potential renal risks associated with specific regimens, particularly those containing abacavir. Alternative antiretroviral options with more favourable renal safety profiles should be explored when appropriate.

## Limitations

Several limitations should be considered when interpreting the results of this study. The cross-sectional nature of the analysis prevents the establishment of causal relationships between renal dysfunction and the identified factors. Longitudinal studies are needed to explore the temporal dynamics of these associations. Moreover, the study was conducted at a single centre, which may limit the generalizability of the findings to broader populations.

In conclusion, this study highlights a substantial prevalence of kidney diseases in HIV-infected children and establishes an association between renal dysfunction and both high viral load and the use of abacavir-based antiretroviral regimens. These findings emphasize the importance of regular renal monitoring in this population and advocate for thoughtful selection of antiretroviral therapies to minimize the risk of renal complications. Further research is warranted to elucidate the underlying mechanisms of nephrotoxicity associated with abacavir and tenofovir and to explore alternative treatment strategies for HIV-infected children.

## Figures and Tables

**Table 1:** Definition of Estimated Glomerular Filtration Rate category 19

Grade	Definition	eGFR (ml/min/1.73m^2^)
1	Normal to increased GFR	≥ 90
2	Mildly reduced GFR	60-89
3a	Mild to Moderately reduced GFR	45-59
3b	Moderate to severely reduced GFR	30-44
4	Severely reduced GFR	15 – 29
5	Kidney failure	< 15
